# Analytical Determination of Selected Volatile Fumigants and Sterilants in Food: Current Status and Future Perspectives

**DOI:** 10.3390/foods15213982

**Published:** 2026-11-06

**Authors:** Marija Kovač Tomas

**Affiliations:** Department of Food Technology, University North, Trg dr. Žarka Dolinara 1, 48000 Koprivnica, Croatia; markovac@unin.hr

**Keywords:** volatile fumigants, pesticide residues, residue definition, inorganic marker ions, gas chromatography, headspace analysis, method validation, food safety monitoring

## Abstract

Volatile fumigants and sterilants remain relevant to the protection of stored commodities and international food trade, yet their residue analysis presents challenges that differ substantially from those encountered for conventional pesticide residues. Their high volatility, chemical reactivity and diverse residue formation pathways complicate sampling, sample preparation, instrumental determination and regulatory interpretation. This review examines compound-specific analytical strategies for ethylene oxide, phosphine, methyl bromide and sulfuryl fluoride. These four representative compounds illustrate distinct residue behaviours involving parent compounds, transformation products, residues derived from fumigant-generating precursors and inorganic marker ions. The discussion focuses on gas chromatography-based methodologies for volatile analytical targets and associated transformation products, alongside complementary analytical approaches, including liquid and ion chromatography for inorganic marker ions. Sampling and sample-preparation challenges, headspace-based methodologies, marker residue determination, method validation and regulatory performance requirements are considered in relation to food safety monitoring. Particular attention is given to the relationship between analytical targets and residue definitions and to the limitations associated with interpreting inorganic marker residues as evidence of fumigant use. Priorities for further development include harmonised validation and sample-handling procedures, improved occurrence and storage-stability data, and fit-for-purpose analytical methods capable of supporting reliable regulatory monitoring.

## 1. Introduction

Modern agricultural production relies on chemical plant protection products to reduce crop losses and support food production for a growing global population. However, the widespread use of pesticides may result in increased exposure of humans and non-target organisms and in the release of these substances into the environment, raising concerns related to food safety and public health [1,2]. Consequently, the monitoring and control of pesticide residues have become a key component of food safety systems worldwide. While regulatory frameworks and analytical monitoring commonly address pesticides applied during primary agricultural production, pesticide residues may also arise from treatments applied during post-harvest handling, storage and transport, as well as from mixing or contamination occurring at subsequent stages of the food supply chain [3,4,5,6]. Among pesticides applied beyond the field stage, fumigants represent a specialised group predominantly used during post-harvest storage and commodity protection [4]. Unlike most conventional pesticides, fumigants act in the vapour phase, facilitating their distribution and penetration within bulk materials and enclosed treatment systems. These properties make fumigants particularly suitable for pest control in stored commodities such as cereals, spices and dried foods [4,7,8]. At the same time, these properties pose significant analytical challenges. Their volatility, potential chemical reactivity and occurrence at low concentration levels in complex food matrices may complicate sample handling, extraction and reliable quantification. In addition, certain compounds may undergo rapid dissipation and chemical transformation within the food matrix, leading to the formation of reaction products that are relevant from both analytical and regulatory perspectives. As a result, conventional multiresidue approaches designed to simultaneously determine a wide range of pesticides may not adequately cover highly volatile fumigants without appropriate modification. Dedicated compound-specific analytical strategies are therefore often required, frequently involving gas chromatography (GC) for volatile analytical targets and implemented in accordance with harmonised validation and quality-control requirements [8,9,10].

Regulatory and analytical attention to fumigant residues increased during 2020–2021, particularly following the detection of ethylene oxide residues in sesame seeds and other plant-derived products. These detections resulted in numerous notifications within the Rapid Alert System for Food and Feed (RASFF) across the European Union (EU). They highlighted the broad distribution of affected products through international supply chains, the analytical challenges associated with the control of ethylene oxide and 2-chloroethanol, and the need for targeted, compound-specific methods capable of meeting regulatory performance criteria [9,11,12]. However, the relevance of fumigants to food and feed monitoring extends beyond ethylene oxide. A recent assessment identified 34 active substances associated with fumigation practices and proposed a shortlist of 13 fumigants of particular relevance for animal feed monitoring, including phosphine-generating compounds, sulfuryl fluoride, hydrogen cyanide, methyl bromide and several historically used halogenated fumigants [5]. These findings highlight the diversity of fumigants and fumigant-generating substances potentially relevant to analytical control and support the need for analytical approaches tailored to their physicochemical and residue properties.

The aim of this review is to examine current analytical approaches for the determination of selected volatile fumigants and sterilants in food. Ethylene oxide, phosphine, methyl bromide and sulfuryl fluoride were selected to represent distinct residue behaviours and analytical targets, and the review is not intended to provide exhaustive coverage of fumigants relevant to the food supply chain. The evidence base was assembled using a narrative approach structured around the four selected compounds rather than a systematic review. Peer-reviewed analytical studies were identified primarily through searches in Google Scholar, with complementary searches in PubMed and ScienceDirect. Searches were conducted from early 2026 through September 2026, using the names of the selected compounds together with terms concerning fumigant residues in food, residue behaviour, analytical determination, sample preparation and method validation. Searches were iterative, with terms adjusted as relevant studies were identified; no single predefined search string was used, and no publication-year restriction was applied. EU legislation was consulted separately through the EUR-Lex online legal information portal, alongside relevant regulatory and technical documents. Sources were selected for their relevance to the occurrence or use of the selected compounds, their residue behaviour or regulatory definitions, and their analytical determination in food matrices. Older publications were retained to provide relevant information not adequately covered in more recent literature.

The discussion covers GC-based methodologies for volatile parent compounds and associated transformation products, together with complementary approaches for inorganic marker ions. Key analytical challenges associated with these compounds, including volatility, chemical reactivity, residue transformation and matrix effects, are considered alongside sampling, sample preparation, instrumental determination and method validation. Recent analytical developments and regulatory considerations are discussed in relation to food safety monitoring and future priorities for fumigant residue analysis. The contribution of this review lies in its cross-compound synthesis, linking residue behaviour and regulatory residue definitions across four selected compounds with analytical method selection and interpretation of results. It also highlights limitations in the available evidence on residue occurrence, storage stability and method validation across food matrices.

## 2. Selected Volatile Fumigants: Properties, Uses and Residue Behaviour

### 2.1. Scope and Classification Rationale

For the purpose of this review, selected volatile fumigants and sterilants are considered together because their analytical relevance in food is closely associated with post-harvest treatment, storage and transport of food commodities [4,8]. Here, fumigants are defined as substances applied or generated in the vapour phase for pest control within enclosed or controlled treatment systems, while sterilants refer to compounds primarily intended for microbial decontamination. Although these terms are conceptually distinct, they may overlap in practical and analytical contexts, as certain substances fulfil both roles depending on their application. Ethylene oxide represents a prominent example because its use as a sterilant may result in a highly volatile parent residue and more persistent reaction products in treated food commodities [9,13]. From an analytical and regulatory perspective, this group of substances cannot be treated as a homogeneous class, as differences in volatility, chemical reactivity and residue behaviour are reflected in compound-specific residue definitions and require tailored analytical strategies [4,8]. The four compounds selected for detailed discussion illustrate contrasting analytical and regulatory residue scenarios, including parent fumigants, transformation products, residues associated with fumigant-generating precursors and inorganic marker ions.

### 2.2. Representative Volatile Fumigants Used in the Food Supply Chain

Volatile fumigants are used in several settings, including soil disinfestation, stored-product and structural pest control, and commodity or quarantine treatments [4,14,15]. Within the agri-food context, the analytical relevance of volatile fumigants and sterilants is linked to their application in enclosed or controlled treatment systems and to the occurrence of parent compounds, transformation products, residues derived from fumigant-generating precursors or marker ions in food commodities [5,8,9,16]. These characteristics distinguish fumigants from most conventional plant protection products and underpin the need for dedicated analytical consideration. The diversity of fumigants currently considered relevant for food and feed safety monitoring is also reflected in differences in their regulatory status and residue behaviour [4,8]. A recent assessment has highlighted that both currently authorised fumigants and historically used compounds may remain relevant from a monitoring perspective and that further analytical method development is required for some substances [5]. Despite their vapour-phase use, volatile fumigants and sterilants differ in their chemical structure, volatility, reactivity and persistence. These differences affect residue formation, transformation pathways and analytical behaviour [4,8]. Their principal applications, residue behaviour, analytical targets and EU residue definitions are summarised in Table 1.

#### 2.2.1. Ethylene Oxide: A Reactive Sterilant and Its Transformation Products

Ethylene oxide is a low-molecular-weight fumigation and sterilisation agent characterised by high vapour pressure and pronounced chemical reactivity [9,13]. From a residue chemistry perspective, ethylene oxide is highly reactive and undergoes chemical transformation within food matrices. Several reaction products may be formed, including ethylene glycol, 2-chloroethanol and 2-bromoethanol, with the latter two being formed through reactions with chloride and bromide ions. In addition, ethylene oxide can react with matrix constituents, leading to the formation of hydroxyethyl adducts, while 2-chloroethanol may participate in esterification reactions. As 2-chloroethanol is more persistent than the parent compound and is not readily removed during aeration, it serves as the principal reaction product included in regulatory residue control [9]. Consequently, the European Union residue definition for ethylene oxide includes both ethylene oxide and 2-chloroethanol, with their sum expressed as ethylene oxide [17,23]. Although the use of ethylene oxide as a plant protection product has been prohibited in the EU since the early 1990s, harmonised maximum residue levels have been established for enforcement purposes [9,23]. The applicable MRLs are generally set at limits of analytical determination and vary according to commodity [17,23].

#### 2.2.2. Phosphine and Phosphine-Generating Compounds

Phosphine (phosphane, PH_3_) is a major fumigant used for stored-product protection. It is widely used for the control of pests in grain storage due to its efficacy, relatively low cost and ability to readily penetrate grain masses [4,8]. A recent EU peer review considered representative post-harvest uses involving indoor fumigation in gas-tight rooms or containers for stored grains, cacao and coffee beans, tree nuts, oilseeds and dried fruits [18]. In practical application within stored-product protection, phosphine may be applied either directly as a gas or generated in situ from solid metal phosphide formulations. The latter are most commonly used for durable commodities and include aluminium phosphide or magnesium phosphide, which release phosphine gas upon contact with moisture. Extensive and long-term reliance on phosphine has contributed to the development of resistance in stored-product pest species, posing a significant challenge to its sustainable use [4,15].

From a regulatory perspective, the residue definition for phosphine encompasses both the parent compound and phosphine-generating substances, namely relevant metal phosphide salts. Residues are therefore defined as the sum of phosphine and phosphine generators, determined and expressed as phosphine [17,18,24]. This definition reflects the predominant use of metal phosphides as precursor formulations and has direct implications for analytical methods in which phosphine may be released from residual phosphides during sample preparation [16,19]. Commodity-specific MRLs are established for this combined residue definition and vary among commodities [17,24].

#### 2.2.3. Methyl Bromide and Sulfuryl Fluoride: Halogenated Fumigants and Inorganic Marker Ions

Halogenated fumigants represent a chemically distinct group of volatile substances characterised by the presence of halogen atoms. Methyl bromide was widely used for several decades because its high volatility and penetration enabled effective pest control over a broad range of treatment conditions. Despite its high volatility, laboratory fumigation studies have demonstrated matrix-dependent persistence of the parent compound, with measurable residues reported for up to ten weeks in nuts and seeds and four weeks in dried fruit under the tested fumigation and airing conditions [25]. However, due to its classification as an ozone-depleting substance under the Montreal Protocol, the use of methyl bromide has been progressively phased out [4,26,27]. At the EU level, methyl bromide is not approved as an active substance for plant protection [5,28]. Under Regulation (EC) No 396/2005, MRLs are not established for methyl bromide itself, whereas commodity-specific MRLs are established for the bromide ion [29]. Although dedicated analytical methods can determine the parent compound [20], EU MRL-based regulatory control is therefore based on the bromide ion. Its analytical interpretation nevertheless requires caution because the measured bromide ion cannot necessarily be attributed specifically to methyl bromide treatment [5,8].

Sulfuryl fluoride, another member of this group, is a broad-spectrum fumigant with high penetration and diffusion capacity and has been adopted as an alternative to methyl bromide for structural and post-harvest fumigation [4]. Following treatment, the parent compound may dissipate, and the fluoride ion represents an additional residue of regulatory relevance [22]. In the EU regulatory framework, sulfuryl fluoride and fluoride ion are retained as separate, tentative residue definitions expressed individually [22,30]. Consequently, analytical monitoring may involve determination of the volatile parent compound or fluoride ion, depending on the residue definition, food matrix and monitoring objective. Interpretation of fluoride ion concentrations requires consideration of natural background levels and other potential sources of fluoride [22]. This dual residue definition illustrates the need for compound- and target-specific analytical strategies rather than treating fluoride ion as a specific or exclusive indicator of sulfuryl fluoride use.

Taken together, the four selected compounds illustrate the diversity of residue behaviour among volatile fumigants, ranging from reactive transformation products to fumigant-generating precursors and inorganic marker ions. These differences have direct implications for analytical strategy selection and regulatory enforcement, underscoring why volatile fumigants are not consistently covered by conventional multiresidue approaches. Reliable implementation of compound-specific analytical strategies also depends on appropriate sampling and sample preparation, which are discussed in the following section.

## 3. Sampling and Sample Preparation Challenges for Volatile Fumigants

Reliable determination of contaminants in foods generally depends on representative sampling and analyte preservation, requirements that are particularly important in the analysis of volatile fumigants and sterilants. Owing to their high vapour pressure and, in some cases, pronounced chemical reactivity, these compounds may be susceptible to losses or transformation prior to determination [4,8,9,19]. Consequently, analytical results may be influenced not only by instrumental performance but also by processes occurring during sample collection, transport, storage and preparation. These pre-analytical challenges are examined below in the context of sampling and sample preparation.

### 3.1. Sampling Strategies

Fit-for-purpose analytical methods must be complemented by representative sampling procedures to ensure reliable pesticide residue analysis. Studies examining conventional, predominantly field-applied pesticide residues have demonstrated considerable variability among individual crop units and samples originating from the same treated lot. Residue distributions may be positively skewed as a result of differences in pesticide application practices, commodity characteristics and post-treatment processes [31,32,33]. Sampling variability may therefore contribute substantially to the overall uncertainty associated with pesticide residue monitoring results [32,33].

The implications of this variability extend beyond analytical measurement and directly influence sampling design and the interpretation of residue monitoring data. Within the European Union, official sampling for pesticide residue control is harmonised through Commission Directive 2002/63/EC, which establishes Community methods of sampling for the determination of pesticide residues in products of plant and animal origin. The objective of the Directive is to obtain representative samples from a lot for the assessment of compliance with established maximum residue limits (MRLs). To achieve this, the Directive provides harmonised definitions of lots, primary samples, bulk samples and laboratory samples, and specifies requirements regarding sample collection, the number of primary samples, minimum sample sizes and sample handling procedures. The Directive also defines criteria for compliance assessment, emphasising the importance of representative sampling in regulatory decision-making. Furthermore, it recognises that MRLs for products of plant origin are based on composite samples derived from multiple units and intended to represent the average residue level within a lot [34]. However, the use of composite samples involves a degree of averaging, which may mask localised contamination and reduce the visibility of extreme residue values occurring in individual crop units [31].

While these principles provide an established framework for conventional pesticide residue monitoring, they do not explicitly address the behaviour of highly volatile compounds. The physicochemical properties of volatile fumigants and sterilants require additional consideration during sampling and sample handling. Unlike conventional contact or systemic pesticides, fumigants are distributed in the vapour phase and their concentrations within food commodities are influenced by diffusion, sorption and desorption processes, and post-treatment aeration [4,8,20]. In addition, residue levels may continue to change during storage and transport as a result of volatilisation and chemical transformation processes, as demonstrated for ethylene oxide, for which evaporation represents the principal route of loss and reactions with matrix constituents may contribute to residue transformation [9].

Although general pesticide sampling principles remain applicable, fumigant-specific information on within-lot variability and sampling uncertainty remains limited. Changes occurring before instrumental analysis may directly influence residue quantification and regulatory assessment [8,9,19]. These considerations provide the basis for understanding the sample preparation challenges associated with volatile fumigants, discussed in the following section.

### 3.2. Sample Preparation for Volatile Fumigant Analysis

While representative sampling is essential for obtaining meaningful residue data, reliable quantification also depends on appropriate sample preparation procedures. For volatile fumigants, particular attention must be paid to factors that may affect analyte recovery and residue stability prior to instrumental analysis.

One of the principal challenges during sample preparation is the prevention of analyte loss. Volatilisation may occur during sample transfer, vessel opening, homogenisation, extraction and storage of standards or sample extracts. Guidance developed for ethylene oxide identifies evaporation and reactions with matrix constituents as major dissipation pathways. For standard solutions, handling at low temperature reduces evaporation losses, while cold vessels should be opened only briefly to limit water condensation. Specifically, ethylene oxide stock and working solutions can be stored in screw-capped glass bottles with Teflon seals in the refrigerator for at least one month or for longer periods in a freezer; diluted calibration solutions should be prepared fresh on the day of analysis [9]. Similarly, phosphine determination requires rapid sample processing and minimisation of handling steps. Following acidification, the vial should be closed immediately, and the interval before instrumental analysis should be controlled because prolonged waiting may result in analyte loss [16,19]. These compound-specific precautions are consistent with general pesticide residue guidance, which recommends low-permeability packaging materials for samples intended for fumigant residue analysis, rapid transport to the laboratory and initiation of analytical procedures as soon as practicable to minimise analyte losses and sample deterioration [10].

In addition to analyte preservation, sample preparation must ensure adequate residue accessibility and representative analytical portions. Milling, grinding and other comminution procedures improve sample homogeneity and extraction efficiency but may affect unstable or volatile residues. Homogenisation therefore presents a particular challenge because it requires a balance between residue accessibility and analyte preservation. Sample heating during grinding may further enhance losses, particularly for highly volatile analytes. For methyl bromide, ultrasonic homogenisation was required to release analyte retained within the commodity and obtain homogeneous analytical portions, but measurable analyte losses occurred during the procedure [35]. Accordingly, low-temperature homogenisation has been recommended when comminution is expected to affect residue stability, while whole units or representative segments may be used when comminution is known to affect fumigant residues and practical alternative procedures are unavailable [10]. More generally, homogenisation conditions should be adapted to the characteristics of the commodity. For high-fat matrices such as nuts and oilseeds, cooling during milling has been recommended to improve homogenisation efficiency and prevent the formation of pasty or insufficiently comminuted material. Studies on sesame have demonstrated that the extraction efficiency of 2-chloroethanol may be strongly influenced by the degree of comminution and extraction conditions. Extraction aids such as stainless-steel balls improve residue accessibility and can reduce the extraction time required to achieve adequate residue recovery [9].

Storage stability represents an additional consideration in fumigant residue analysis. Owing to their volatility and potential chemical reactivity, residue concentrations may change during storage of food samples, extracts and standard solutions. This risk may be increased by prolonged storage, inappropriate temperatures and repeated handling, potentially affecting analyte recovery and quantitative reliability [9,19]. Stability under the intended sample and extract storage conditions should therefore be established or verified, and acceptable intervals between sampling, preparation and analysis should be defined where relevant; however, the available information does not allow a direct comparison of storage-temperature effects among the fumigants and food matrices considered [9,10,19].

The challenges associated with analyte preservation have strongly influenced the development of sample preparation methodologies for volatile fumigants. For analytical targets amenable to vapour-phase isolation, headspace-based approaches can be particularly suitable, as they can reduce direct manipulation of samples and extracts and exploit analyte partitioning into the vapour phase [8]. Static and dynamic headspace techniques provide different options for gas-phase sampling, while purge-and-trap systems enable additional analyte enrichment before instrumental determination. Headspace solid-phase microextraction (HS-SPME) further combines sampling, extraction and preconcentration in a single solvent-free step [8,36]. The applicability of HS-SPME to the simultaneous determination of multiple volatile fumigants in food matrices has been demonstrated, with method performance depending on SPME fibre selection, equilibration conditions and the time-dependent sorption behaviour of individual fumigants [20].

Despite the advantages of gas-phase sampling techniques, solvent-based extraction remains relevant for less volatile transformation products and for analytical targets that must be released efficiently from the matrix. The appropriate sample-preparation strategy therefore depends not only on the original fumigant but also on the physicochemical properties of the residue being determined and its interaction with the commodity matrix [8,9]. The analytical application and performance of these approaches are discussed further in Section 4.1.

Overall, successful fumigant residue analysis requires a balance between preserving analyte integrity and ensuring representative sample preparation and efficient residue release from the matrix. Failure to meet either requirement may compromise quantitative results despite adequate instrumental sensitivity and selectivity.

## 4. Analytical Approaches for Selected Volatile Fumigants

### 4.1. GC-Based Determination of Volatile Fumigants and Associated Transformation Products

The analysis of volatile fumigants requires analytical approaches that account for the possibility of analyte isolation either in the liquid phase or directly in the vapour phase. The volatility of fumigants presents both analytical challenges and opportunities. While highly volatile compounds may be difficult to retain during sample handling and are not always compatible with conventional clean-up procedures, their transfer into the vapour phase can enable their isolation from complex food matrices. Vapour-phase analysis can provide an effective clean-up step by reducing matrix interferences, minimising solvent-related contamination and allowing rapid determination by gas chromatography. These characteristics have strongly influenced analytical method development and made GC a central technique for the determination of volatile fumigant residues [8,36]. The occurrence and intensity of matrix interferences from natural sample constituents may vary with the determination system [10].

Fumigant isolation may involve solvent extraction or gas-phase sampling approaches, including headspace and purge-and-trap techniques [8]. These approaches are not mutually exclusive but provide a useful framework for understanding current analytical methodologies. Among them, headspace techniques are particularly applicable to volatile fumigants because they exploit analyte partitioning into the vapour phase while reducing the transfer of non-volatile matrix components into the analytical system [8,36].

Headspace techniques involve the transfer of analytes from the sample matrix into a closed gaseous phase prior to chromatographic determination. In contrast to extraction-based procedures, which transfer residues into a liquid phase, headspace methodologies utilise vapour-phase partitioning to facilitate analyte isolation from matrix components. Static headspace (HS) sampling represents the simplest approach, whereas dynamic headspace (DHS) continuously sweeps volatile compounds from the sample headspace and concentrates them on an adsorbent trap prior to thermal desorption and analysis. Additional enrichment can be achieved through headspace solid-phase microextraction (HS-SPME), which combines sampling, extraction and preconcentration into a single solvent-free step [8,36]. Du et al. [20] demonstrated simultaneous determination of eight volatile fumigants in cereals, oilseeds, nuts and dried fruit using HS-SPME-GC-MSD, highlighting the importance of SPME fibre selection, equilibration conditions and analyte sorption behaviour for reliable residue quantification across different food matrices. However, analytical performance varied among the fumigants, with sulfuryl fluoride showing poorer precision under the tested conditions than the other compounds in the method [20]. This result warrants caution when interpreting quantitative results for sulfuryl fluoride obtained using this method but does not by itself establish that the method is unsuitable for routine use. Purge-and-trap techniques differ from equilibrium-based static headspace analysis in that they aim for greater transfer of fumigants into the vapour phase [8,36].

In routine food control, GC coupled to mass spectrometry (GC-MS) and tandem mass spectrometry (GC-MS/MS) are widely applied for the determination of volatile analytical targets. While GC-MS remains suitable for the determination of individual fumigants, GC-MS/MS can offer enhanced selectivity and sensitivity, particularly when low residue levels must be determined in complex food matrices [9,36]. The practical application of these analytical concepts is evident in the compound-specific approaches required for the selected fumigants, reflecting differences in their residue behaviour, regulatory residue definitions and analytical requirements.

Ethylene oxide illustrates the use of complementary solvent-extraction and gas-phase enrichment strategies. Owing to the volatility of ethylene oxide and the low residue levels relevant to regulatory control, analytical determination requires sensitive and selective methodologies capable of addressing both the parent compound and 2-chloroethanol, the principal reaction product included in the EU residue definition. GC-MS/MS determination following modified QuEChERS- or QuOil-based extraction procedures has been described, with matrix-matched calibration and isotopically labelled internal standards used where appropriate [9]. By contrast, the German official method for spices (BVL L 53.00-1) determines the combined residue, expressed as ethylene oxide, by converting 2-chloroethanol back to ethylene oxide and subsequently derivatising the resulting ethylene oxide to 2-iodoethanol for GC analysis [37,38]. More recently, dynamic headspace GC-MS/MS methodologies have also been proposed for the analysis of acetonitrile extracts, demonstrating the applicability of gas-phase enrichment techniques for the simultaneous determination of both analytes in complex dry food commodities. For ethylene oxide, matrix-derived acetaldehyde has been reported as a potential interference; primary–secondary amine clean-up reduced its signal in selected matrices but was not required for sesame [39]. Other gas-phase enrichment strategies include SPME-GC-MS methods developed specifically for low-viscosity hydroxypropyl methylcellulose used in dietary supplement capsules. The method employed standard addition and demonstrated the potential for automated determination, while also identifying contamination from plastic containers and analytical carry-over as possible sources of error [40]. An automated cryogen-free headspace-trap GC-MS method with multi-step enrichment enabled simultaneous determination of both analytes without solvent extraction or derivatisation [41]. Together, these approaches show how analytical workflows can employ different modes of analyte isolation while addressing both components of the EU residue definition [9].

In contrast, phosphine residue analysis involves determination of phosphine released into the vial headspace, generally following sample acidification. Because acidification may also release phosphine from residual metal phosphides, the analytical result can include both phosphine present in the sample and phosphine generated from precursor compounds during sample preparation. Because of its extreme volatility and the typically low residue levels encountered in treated commodities, headspace-based methodologies are widely applied. Both regulatory guidance and published applications describe the use of HS-GC-MS methods capable of achieving limits of quantification (LOQs) below 1 µg kg^−1^, provided that acid addition, vial closure and the interval before analysis are carefully controlled [16,19]. A related HS-GC-MS method employing 5% sulfuric acid and heated headspace extraction was evaluated for phosphine residues in wheat and maize, with a reported LOQ of 1.38 µg kg^−1^ and relative standard deviations (RSDs) of 7.6% and 6.3%, respectively, at a fortification level of 0.01 mg kg^−1^ [42]. These methods therefore correspond to the regulatory definition covering the sum of phosphine and phosphine generators, expressed as phosphine, but do not necessarily distinguish between previously free and analytically generated phosphine [19,24].

The parent compound methyl bromide can be determined using dedicated GC-based methods, including automated headspace GC applied to stored foods and the HS-SPME-GC-MSD approach described for simultaneous determination of multiple fumigants [20,25]. The complementary determination and regulatory interpretation of bromide ion are discussed in Section 4.2.

Sulfuryl fluoride provides an additional example of how analytical strategies may be influenced by residue behaviour. When the parent fumigant is the analytical target, GC-based methodologies remain suitable, and HS-GC-MS methods have been reported for its determination in cereals, nuts, pulses and other dry commodities [21]. A complementary method based on water extraction in an airtight blender jar and HS-GC with electron capture detection and GC-MS confirmation provided recoveries of 74.1–108.9% and RSDs of 0.5–6.8% in agricultural products [43]. However, sulfuryl fluoride differs from phosphine because the fluoride ion also constitutes a separate analytical target under the tentative EU residue definitions [22,30]. Methods for its determination and the limitations affecting interpretation of the resulting data are discussed in Section 4.2.

### 4.2. Determination and Interpretation of Inorganic Marker Ions

In contrast to many conventional pesticides, where enforcement is typically based on determination of the parent compound or a limited number of metabolites, fumigants exhibit markedly different residue behaviours. Depending on the compound, regulatory residue definitions may target the parent fumigant, a transformation product or a relevant inorganic marker ion. These differences have important analytical implications because the selected analytical target strongly influences the choice of methodology, sample preparation strategy and instrumental technique [5,8,9].

Methyl bromide provides a representative example of the distinction between direct determination of a parent fumigant and regulatory monitoring based on an inorganic marker ion. Dedicated GC-based methods can determine the parent compound, whereas the EU residue definition and associated MRLs are established for the bromide ion [17,20,29]. Accordingly, analytical control can be performed using ion chromatography-tandem mass spectrometry (IC-MS/MS) or liquid chromatography-tandem mass spectrometry (LC-MS/MS) methodologies rather than conventional GC-based determination of the parent fumigant. For the bromide ion, analytical workflows may be based on the QuPPe (Quick Method for the Analysis of Highly Polar Pesticides) approach, combining dedicated sample preparation with IC-MS/MS or LC-MS/MS determination [44]. QuPPe extraction combined with capillary electrophoresis-tandem mass spectrometry (CE-MS/MS) has also recently been validated for the bromide ion in selected food matrices, although its wider applicability across commodities remains to be established [45]. Interpretation of bromide-ion results requires caution because the measured ion is not specific to methyl bromide treatment and its origin cannot necessarily be determined from the analytical result alone [8].

Fluoride ion represents a related but analytically distinct case. For sulfuryl fluoride, the parent compound and fluoride ion are retained as separate, tentative residue definitions. A method based on a fluoride ion-selective electrode has been validated for several high-oil, dry and dried-fruit matrices, whereas method availability and validation remain incomplete for a number of other commodity groups. In addition, natural background concentrations and fluoride originating from other sources may limit the extent to which a measured fluoride concentration can be attributed specifically to sulfuryl fluoride treatment [22]. More recently, capillary electrophoresis coupled with capacitively coupled contactless conductivity detection (CE-C4D) was applied to fluoride determination in corn, rice and wheat before and after sulfuryl fluoride treatment and compared with fluoride ion-selective electrode analysis. The method provided a mean recovery of 84% with an RSD of 12% at 1.0 mg kg^−1^ and a detection limit of 0.3 mg kg^−1^. Additional method development was considered necessary for high-sugar and high-fat commodities because of potential matrix interferences [46]. Together, these examples demonstrate that the determination and interpretation of inorganic marker ions depend on the individual ion, food matrix, applicable residue definition and monitoring objective. Methods validated for one marker ion or commodity group should not be assumed to be directly applicable to another. Representative analytical methods for the analytical targets discussed in Section 4.1 and Section 4.2 are summarised in Table 2.

The analytical approaches summarised in Table 2 vary in terms of instrumental determination, analytical target, sample-preparation requirements and validation design. The reported performance data are therefore method- and matrix-specific and do not provide a uniform basis for direct comparison across the selected analytical targets.

The distribution of analytical evidence across these targets is also uneven. Within the literature considered in this review, methods for ethylene oxide and 2-chloroethanol, as well as phosphine, are represented by several GC-based workflows and matrix-specific performance data. Peer-reviewed evidence remains more limited for methyl bromide, sulfuryl fluoride and their associated inorganic marker ions, although recent studies have begun to expand the available methodological options. For these targets, the available evidence also includes multianalyte methods, single-residue protocols and regulatory assessments. This does not necessarily indicate limited monitoring relevance, but it restricts direct comparison of analytical performance and demonstrates the continuing importance of official and regulatory methods where published validation data remain sparse. Further analytical development should therefore prioritise validation using incurred residues, interlaboratory evaluation, storage-stability assessment and broader commodity-group coverage rather than improvements in instrumental sensitivity alone.

### 4.3. Method Validation and Regulatory Performance Requirements

Because volatile fumigants exhibit compound- and target-specific analytical characteristics, methods used for regulatory monitoring and food safety assessment must be shown to consistently fulfil predefined analytical performance requirements. Validation should also give particular attention to analyte integrity throughout the analytical workflow, from sample preparation to instrumental determination [8,9,10,19].

Within the European Union, analytical methods for pesticide residue determination are validated according to the performance criteria specified in the current SANTE guidance document, including requirements related to specificity, trueness, precision, limits of quantification, matrix effects and quality control [10].

The practical implementation of these validation requirements varies according to the analytical concept employed. For conventional GC-based methods targeting parent fumigants or transformation products, validation studies typically focus on achieving adequate sensitivity, recovery and precision, as well as evaluating and, where necessary, compensating for matrix effects, while minimising analyte losses during sample preparation and instrumental analysis. Depending on the analyte and matrix, reliable quantification may require matrix-matched calibration, standard addition or isotopically labelled internal standards [9,19]. For example, a recently developed GC-MS/MS method for the simultaneous determination of ethylene oxide and 2-chloroethanol demonstrated compliance with the applicable SANTE performance criteria, achieving a limit of quantification of 0.01 mg kg^−1^, recoveries of 74–120%, and RSD values below 12% across diverse food matrices [47].

The available validation evidence is similarly heterogeneous. Methods reported for ethylene oxide and 2-chloroethanol have been evaluated across multiple food matrices, whereas validation of phosphine methods is complicated by the limited suitability of conventional fortification experiments and the need to use incurred samples or alternative calibration strategies [16,48]. For inorganic marker ions, method coverage remains uneven: QuPPe-based LC-MS/MS and IC-MS/MS procedures provide performance data for the bromide ion in several commodity groups, whereas CE-MS/MS validation has so far been demonstrated in two selected matrices. For fluoride ion, validated data are reported for certain dry, high-oil and dried-fruit matrices, but analytical coverage remains incomplete for other commodity groups [22,44,45]. These differences should be considered when assessing whether a method is fit for a particular monitoring purpose and when defining the corresponding validation requirements.

For highly volatile fumigants such as phosphine, method validation may require additional adaptations beyond conventional pesticide residue protocols. Conventional fortification and recovery experiments may not adequately reproduce incurred or aged phosphine residues, and quantitative determination may require procedural calibration using a suitable blank matrix or standard addition [8,16,19,48]. A recent HS-GC-MS method development study has demonstrated that optimisation of headspace conditions, sample acidification, immediate vial closure and careful evaluation of matrix effects are important for obtaining reliable analytical performance [48]. The observations reported by Lin et al. are consistent with earlier EURL recommendations highlighting the importance of prompt sample processing and minimising analyte losses throughout the analytical workflow [19]. Two interlaboratory comparisons of a headspace method based on sample heating in 5% sulfuric acid showed considerable between-laboratory variation despite good within-laboratory reproducibility, resulting in an estimated measurement uncertainty of approximately 50% and indicating the need for further proficiency testing [49]. At the regulatory level, a recent EU peer review reported GC-NPD methods with an LOQ of 0.01 mg kg^−1^ for dry and high-water-content commodities and coffee and a GC-MS/MS method with the same LOQ for high-acid and high-oil-content commodities. However, an independent laboratory validation was not available for the latter method [18]. Validation should therefore include the relevant sample-preparation and headspace conditions rather than being limited to instrumental performance [10].

Storage stability requires particular attention during validation of analytical methods for volatile fumigants. Losses resulting from volatilisation, chemical transformation or inappropriate storage conditions may influence analytical recovery, repeatability and quantitative performance. Stability under the intended sample and extract storage conditions should therefore be evaluated or verified where relevant as part of method validation [9,10,19].

These validation considerations are central to the generation and interpretation of residue data used in regulatory monitoring and food safety assessment [10].

## 5. Future Perspectives and Implications for Food Safety Monitoring

The evidence reviewed in the preceding sections shows that volatility, chemical transformation and diverse residue formation pathways can affect multiple stages of the analytical workflow [4,8,9,19].

The implications of fumigant residue behaviour extend beyond analytical determination to regulatory monitoring and interpretation of the resulting data. For the selected fumigants, enforcement residue definitions may encompass transformation products or inorganic marker ions rather than the parent compound alone. Examples include the combined residue definition for ethylene oxide and 2-chloroethanol, the bromide ion definition associated with methyl bromide control, and the separate tentative residue definitions for sulfuryl fluoride and fluoride ion [9,22,29,30]. The interpretation of the results must account for the relevant residue formation pathways and, in the case of inorganic marker ions, potential background contributions and the extent to which the measured residue can be attributed to the original fumigant.

From a food safety perspective, the distinction between parent compounds and transformation products is particularly important because residue definitions used for enforcement may not encompass all compounds relevant to dietary risk assessment [50]. Improved information on the occurrence and toxicological relevance of fumigant-derived residues would support more reliable exposure assessment and interpretation of monitoring data.

Evidence gaps also concern the occurrence of fumigant residues and differences in reported uses across commodities and treatment settings. While phosphine, sulfuryl fluoride and ethylene oxide have received regulatory attention, systematic occurrence information remains sparse for several fumigants identified as potentially relevant to food and feed monitoring. This lack of harmonised occurrence information complicates the establishment of evidence-based monitoring priorities and may hinder effective allocation of analytical resources. Future monitoring programmes may therefore benefit from prioritisation approaches that focus analytical resources on fumigants considered most relevant based on regulatory and toxicological criteria and available evidence of occurrence [5].

For routine monitoring, harmonised sample-handling procedures and storage-stability studies should complement instrumental improvements. Consistent validation protocols would further improve comparability of results among laboratories and monitoring programmes [8,9,10,19].

At the method level, priorities include automation of headspace-based procedures, development of robust multi-residue workflows for routine monitoring, and reduced sample and solvent consumption, supported by fit-for-purpose validation across representative food matrices [8,20,36]. In the broader field of food contaminant analysis, capillary electromigration techniques have been investigated as approaches requiring low consumption of samples and reagents [51]. Their potential relevance to the analysis of inorganic marker ions considered in fumigant residue monitoring is supported by the application of QuPPe extraction combined with CE-MS/MS to bromide ion in selected food matrices and CE-C4D determination of fluoride ion in sulfuryl-fluoride-treated grain [45,46], although wider applicability across commodity groups and matrix types remains to be established. High-resolution and non-target screening techniques may provide complementary information on pesticide metabolites and transformation products [52], but their applicability to the selected fumigants and their transformation products remains limited.

The available evidence indicates that volatile fumigants are likely to remain relevant to food safety monitoring because of their continued use in global commodity storage and trade, diverse residue behaviour and compound-specific analytical challenges [4,5]. Future monitoring strategies should integrate evidence on residue occurrence, analytical feasibility and toxicological relevance.

## 6. Conclusions

Volatile fumigants and sterilants pose a distinct set of analytical challenges within pesticide residue analysis owing to their high volatility, chemical reactivity and diverse residue behaviour. Their reliable determination requires consideration of instrumental performance together with representative sampling, appropriate sample-handling, analyte preservation and adequate method validation.

The examples of ethylene oxide, phosphine, methyl bromide and sulfuryl fluoride demonstrate that analytical workflows are shaped by residue behaviour and regulatory residue definitions. Depending on the compound and monitoring objective, the analytical target may be the parent fumigant, a transformation product, a residue derived from a fumigant-generating precursor or an inorganic marker ion. The interpretation of inorganic marker ions requires particular caution because their presence cannot necessarily be attributed specifically to treatment with the corresponding fumigant.

These differences require compound- and matrix-specific analytical methods supported by appropriate calibration and adequate validation. However, the available analytical evidence remains uneven across targets, particularly regarding validation using incurred residues, interlaboratory evaluation and commodity-group coverage. Further progress will depend on improved occurrence and storage-stability data and greater harmonisation of sample-handling and method validation procedures.

## Figures and Tables

**Table 1:** Representative applications, residue behaviour, analytical targets and EU residue definitions for selected volatile fumigants and sterilants in food.

Compound	Representative Application	Principal Residue Behaviour	Analytical Targets	EU Residue Definition Under Regulation (EC) No 396/2005 [17]	Main Interpretative Implication
Ethylene oxide	Fumigation of dry foods, including herbs, spices, nuts and oilseeds, for the control of insects and microorganisms [5,9]	The parent compound dissipates through evaporation and reactions within the food matrix, whereas 2-chloroethanol is less readily removed during aeration [9]	Ethylene oxide and 2-chloroethanol	Sum of ethylene oxide and 2-chloroethanol, expressed as ethylene oxide	For compliance assessment, ethylene oxide and 2-chloroethanol are combined, with the result expressed as ethylene oxide [9]
Phosphine	Fumigation of stored cereals and other commodities in enclosed storage facilities or gas-tight containers [4,18]	Residues may comprise phosphine retained in the commodity and residual metal phosphides capable of releasing phosphine [8]	Phosphine released from the sample	Sum of phosphine and phosphine generators, expressed as phosphine	The measured result may include phosphine retained in the sample and phosphine generated from residual metal phosphides during sample preparation [8,19]
Methyl bromide	Quarantine and pre-shipment treatment and historical commodity fumigation [4,5]	The parent compound is highly volatile, while the bromide ion represents an inorganic residue relevant to regulatory control [8]	Bromide ion; the parent compound can also be determined using dedicated methods [20]	Bromide ion	Bromide-ion detection cannot necessarily be attributed specifically to methyl bromide treatment [5,8]
Sulfuryl fluoride	Structural and commodity fumigation [4,21]	The volatile parent compound may dissipate following treatment, and fluoride ion represents an additional residue of regulatory relevance [21,22]	Sulfuryl fluoride; fluoride ion	Sulfuryl fluoride; fluoride ion (separate tentative residue definitions)	Measured fluoride concentrations may include natural background and contributions from sources other than sulfuryl fluoride treatment [22]

Note: The European Commission’s EU Pesticides Database provides searchable information on current and historical MRLs, with links to relevant legislation.

**Table 2:** Representative analytical approaches and reported performance for selected volatile fumigants and associated analytical targets in food.

Analytical Target	Representative Matrices	Sample Preparation or Analyte Isolation	Instrumental Determination	Reported Analytical Performance	Main Analytical Consideration	Reference
Ethylene oxide and 2-chloroethanol	Sesame, cumin, wheat, tea, spices, herbs, dehydrated fruits, food additives, grapes and tomatoes	Acetonitrile extraction at <10 °C followed by dispersive solid-phase extraction with primary–secondary amine and, depending on the matrix, C18 sorbents	PTV-GC-MS/MS	LOQ of 0.01 mg kg^−1^ for both analytes; recoveries of 74–120%; RSD < 12%; interlaboratory reproducibility evaluated in six laboratories	Controlled extraction temperature and clean-up were used to minimise matrix effects	[47]
Ethylene oxide and 2-chloroethanol	Cumin, ashwagandha, chilli powder, turmeric powder, guar gum, locust bean gum, ginger powder and sesame	Extraction of 4 g of sample with 10 mL of acetonitrile; primary–secondary amine clean-up where required; dynamic headspace sampling	DHS-GC-MS/MS	Recoveries of 70–120% with RSD ≤ 15% at 0.01, 0.02 and 0.05 mg kg^−1^; reproducibility evaluated in two laboratories	Primary–secondary amine clean-up reduced the matrix-derived acetaldehyde signal in selected matrices but was not required for sesame	[39]
Ethylene oxide and 2-chloroethanol	Low-viscosity hydroxypropyl methylcellulose used in dietary supplement capsules	Matrix-specific solvation and solid-phase microextraction; quantification by standard addition	SPME-GC-MS	LODs of 6.7 µg kg^−1^ for ethylene oxide and 12 µg kg^−1^ for 2-chloroethanol	The procedures were developed specifically for the solvation properties of the matrix; contamination from plastic containers and analytical carry-over were identified as possible sources of error	[40]
Phosphine	Cereals, cereal products, spices, oilseeds, legumes, nuts and other dried foods	Ground coarse-granular commodities: water followed by 10% sulfuric acid; granular commodities: 5% sulfuric acid; immediate vial closure	HS-GC-MSD	LOQ of 0.1 µg kg^−1^; RSD of 5.8% at 42 µg kg^−1^	Matrix-dependent signal quenching requires standard addition or procedural calibration using a similar matrix for accurate quantification	[16]
Phosphine	Wheat and yellow maize	Extraction with 5% sulfuric acid in a gastight vial followed by heated headspace extraction	HS-GC-MS	LOD and LOQ of 0.46 and 1.38 µg kg^−1^, respectively; RSDs of 7.6% in wheat and 6.3% in maize at 0.01 mg kg^−1^	The procedure reduced the number of analytical steps and was evaluated using fortified and fumigated cereal samples	[42]
Phosphine	Rice, oats, dried corn, white and black sesame, fresh onions, raisins, dried cabbage, almonds, chilli powder, Sichuan pepper and cocoa	Dry-ice-cooled homogenisation; addition of 7.5 mL water and 7.5 mL 10% sulfuric acid to 1 g of sample, providing a final acid concentration of 5%; immediate vial closure	HS-GC-MS	LOQ of 0.005 mg kg^−1^; correlation coefficients > 0.99 in 12 matrices; intraday and interday CVs of 17% for an incurred sesame sample	Signal suppression was observed in 11 of the 12 tested matrices. A standard curve was used for initial estimation, followed by standard addition for quantitative determination; validation was performed using an incurred sample because conventional recovery experiments were considered unsuitable	[48]
Eight volatile fumigants, including ethylene oxide, phosphine, methyl bromide and sulfuryl fluoride	Wheat, oats, canola, almonds and sultanas	Equilibration of the sample in a sealed flask followed by headspace solid-phase microextraction	HS-SPME-GC-MSD	LODs of 0.03–1.99 ng g^−1^; RSDs ≤ 8.7% for most fumigants at 3 ng g^−1^; RSD of 39.7% for sulfuryl fluoride	Fibre selection, extraction conditions and fumigant sorption behaviour influenced analytical performance; performance was poorer for sulfuryl fluoride	[20]
Sulfuryl fluoride	Cereals, pulses, tree nuts and other dry commodities	Granular samples treated with 5% sulfuric acid; powdered samples wetted with water and treated with 10% sulfuric acid in a sealed headspace vial	HS-GC-MSD	Recovery of 94% for soy flour containing 0.021 mg kg^−1^; calibration linear to at least 50 µg kg^−1^; sensitivity sufficient to monitor 0.01 mg kg^−1^	Matrix effects may occur; matrix-matched calibration or, preferably, standard addition is recommended	[21]
Bromide ion	Foods of plant origin from different commodity groups	Water adjustment followed by extraction with acidified methanol; additional preparation depends on the commodity	LC-MS/MS or IC-MS/MS	QuPPe provides method- and commodity-specific performance data rather than a single generally applicable LOQ	In LC-MS/MS, bromide cannot undergo MS/MS fragmentation and is measured using parent/parent transitions. Phosphoric and phosphonic acids may interfere under particular chromatographic conditions	[44]
Bromide ion	Lemon and milk	QuPPe extraction followed by fivefold dilution of the extract	CE-MS/MS	At 5 mg kg^−1^, average recovery of 97% in both matrices, with RSDs of 11% in lemon and 9% in milk	Validation of bromide was performed in two selected matrices; bromide was determined without an isotopically labelled internal standard	[45]
Fluoride ion	High-oil and dry matrices and dried fruits	Not specified in the assessment	Fluoride ion-selective electrode	The assessment reports validated LOQs of 0.05–5 mg kg^−1^, depending on the matrix	Validated methods were not available for several other commodity groups; naturally occurring fluoride must be considered when interpreting results	[22]

Note: Validation parameters are reported as provided in the original studies; inconsistent reporting limits direct comparison across all methods. Abbreviations: C18, octadecylsilane sorbent; CE-MS/MS, capillary electrophoresis–tandem mass spectrometry; CV, coefficient of variation; DHS, dynamic headspace; GC-MS, gas chromatography–mass spectrometry; GC-MS/MS, gas chromatography–tandem mass spectrometry; GC-MSD, gas chromatography–mass-selective detection; HS, headspace; IC-MS/MS, ion chromatography–tandem mass spectrometry; LC-MS/MS, liquid chromatography–tandem mass spectrometry; LOD, limit of detection; LOQ, limit of quantification; MS/MS, tandem mass spectrometry; PTV, programmed-temperature vaporisation; QuPPe, Quick Method for the Analysis of Highly Polar Pesticides; RSD, relative standard deviation; SPME, solid-phase microextraction.

## Data Availability

No new data were created or analysed in this study. Data sharing is not applicable to this article.
